# Kidney Disease in Ankylosing Spondylitis: a case series and review of the literature

**DOI:** 10.1590/2175-8239-JBN-2022-0008

**Published:** 2022-05-13

**Authors:** Ana Cunha Rodrigues, Joana Cristóvão Marques, Marina Reis, Mário Góis, Helena Sousa, Fernando Nolasco

**Affiliations:** 1 Centro Hospitalar Tondela/Viseu, Departamento de Nefrologia, Viseu, Portugal.; 2 Centro Hospitalar de Lisboa Central, Hospital Curry Cabral, Departamento de Nefrologia, Lisboa, Portugal.; 3 Centro Hospitalar Vila Nova de Gaia/Espinho, Departamento de Nefrologia, Vila Nova de Gaia, Portugal.; 4 Centro Hospitalar de Lisboa Central, Hospital Curry Cabral, Laboratório de Morfologia Renal, Lisboa, Portugal.

**Keywords:** Ankylosing Spondylitis, Kidney Diseases, Glomerulonephritis, Interstitial Nephritis, Amyloidosis, Biopsy, Espondilite Anquilosante, Doenças Renais, Glomerulonefrite, Nefrite Intersticial, Amiloidose, Biópsia

## Abstract

**Background:**

Kidney disease is a rare manifestation of ankylosing spondylitis (AS) and its pathological alterations remain poorly described. The aim of this study was to investigate the clinical presentation and pathological alterations on kidney biopsy of AS patients and review and discuss the current literature on the issue.

**Methods:**

We retrospectively studied the clinical presentation and kidney pathological alterations of 15 Caucasian AS patients submitted to kidney biopsy between October 1985 and March 2021.

**Results:**

Patients were predominantly male (66.7%) with median age at the time of kideney biopsy of 47 years [IQR 34 - 62]. Median serum creatinine at presentation was 1.3 mg/dL [IQR 0.9 - 3] and most patients also had either proteinuria (85.7%) and/or hematuria (42.8%). The most common indication for kidney biopsy was nephrotic syndrome (33.3%), followed by acute or rapidly progressive kidney injury (20%) and chronic kidney disease of unknown etiology (20%). Chronic interstitial nephritis (CIN) (n=3) and AA amyloidosis (n=3) were the most common diagnosis. Others included IgA nephropathy (IgAN) (n=2), focal segmental glomerulosclerosis (n=2), membranous nephropathy (n=1), and immune complex-mediated membranoproliferative glomerulonephritis (IC-MPGN)(n=1).

**Conclusions:**

We present one of the largest series of biopsy-proven kidney disease in Caucasian AS patients. We found a lower prevalence of IgAN than previously reported in Asian cohorts. We found a higher prevalence of CIN and a lower prevalence of AA amyloidosis than that described in previous series of Caucasian patients. We also present the first case of AS-associated IC-MPGN.

## Resumo

**Antecedentes:**

A doença renal é uma manifestação rara de espondilite anquilosante (EA) e as suas alterações patológicas permanecem pouco descritas. O objetivo deste estudo foi investigar a apresentação clínica e alterações patológicas na biópsia renal de doentes com EA bem como rever e discutir a literatura atual sobre o assunto.

**Métodos:**

Estudamos retrospectivamente a apresentação clínica e alterações patológicas renais de 15 doentes caucasianos com EA submetidos a biópsia renal entre Outubro de 1985 e Março de 2021.

**Resultados:**

Os doentes eram predominantemente homens (66,7%) com idade mediana no momento da biópsia de 47 anos [IIQ 34 - 62]. A creatinina sérica mediana na apresentação foi de 1,3 mg/dL [IIQ 0,9 - 3] e a maioria dos pacientes apresentava também proteinúria (85,7%) e/ou hematúria (42,8%). A indicação mais comum para biópsia renal foi a síndrome nefrótica (33,3%), seguida de lesão renal aguda ou rapidamente progressiva (20%) e doença renal crónica de etiologia desconhecida (20%). A Nefrite intersticial crónica (NIC) (n=3) e a amiloidose AA (n=3) foram os diagnósticos mais comuns. Outros incluíram nefropatia por IgA (NIgA) (n=2), glomeruloesclerose segmentar focal (n=2), nefropatia membranosa (n=1) e glomerulonefrite membranoproliferativa mediada por imunocomplexos (GNMP-IC) (n=1).

**Conclusões:**

Apresentamos uma das maiores séries de doenças renais comprovadas por biópsia em doentes caucasianos com EA. Encontramos uma prevalência de NIgA menor do que a relatada anteriormente em coortes asiáticas. Encontramos uma maior prevalência de NIC e uma prevalência menor de amiloidose AA do que a descrita em séries anteriores de pacientes caucasianos. Também apresentamos o primeiro caso de GNMP-IC associada à EA.

## Introduction

Ankylosing spondylitis (AS) is an autoimmune-mediated axial spondyloarthropathy that affects primarily spinal and sacroiliac joints. It has a male predominance and its onset peaks in early adulthood. The most common manifestations include inflammatory back pain and progressive spinal rigidity leading to fusion of the spine and immobility^1^. Patients can also experience peripheral arthritis as well as extra-articular manifestations, being the most common acute anterior uveitis (26%), psoriasis (9%), and inflammatory bowel disease (7%)^2^. AS has been associated with an increased risk of kidney disease compared to the general population, which may be an overlooked extra-articular manifestation in AS patients^3^. The exact mechanism of kidney disease remains poorly understood as kidney biopsy results are only rarely reported. We present a case series of 15 Caucasian patients with AS submitted to kidney biopsy and provide a review and discussion of the current literature on renal manifestations of AS.

## Methods

We retrospectively studied renal biopsies of 15 patients with the diagnosis of AS received at our laboratory from October 1985 to March 2021. Histologic diagnosis was reviewed, as were serum creatinine (sCr), urinalysis, and 24-h proteinuria at the time of biopsy. When available, other relevant clinical data were collected.

Proteinuria was defined as protein urinary excretion of more than 300 mg/24h and hematuria as presence of more than five erythrocytes per high power field (400x) on microscopic urine examination. Nephrotic syndrome was assumed whenever serum albumin was <3.5 g/dL with protein excretion of >3.5 g/24h.

Renal biopsy specimens were examined by light microscopy (LM) and immunofluorescence (IF). LM analysis was performed after staining with hematoxylin and eosin (HE), periodic acid Schiff (PAS), periodic acid methenamine silver (PAM), Masson’s trichrome, and Congo-red. IF was performed in frozen sections (or in paraffin fragments after protease digestion as needed) using fluorescein isothiocyanate conjugated antisera to human immunoglobulins (IgG, IgA, IgM), light chains (λ and κ), complement (C1q, C3), and fibrinogen. Amyloid substance was characterized using immunofluorescence or immunochemistry staining for SAA or κ and λ light chains.

## Results

### Baseline Characteristics of the Population and Clinical Features at Presentation

All patients were Caucasian and the majority (68%) was male with median age of 47 years (interquartile range [IQR] 34 to 62 years) and 10 years of median duration of the disease at the time of kidney biopsy (IQR 6 to 15 years). Only one patient was known to have a previous extra-articular manifestation (cutaneous psoriasis) and most patients had been treated with non-steroidal anti-inflammatory drugs (NSAID) (n=5, 33%) and/or Sulfasalazine (n=5, 33%) for AS. Median sCr at presentation was 1.3 mg/dL (IQR 0.9 to 3 mg/dL), mean estimated glomerular filtration rate (CKD-EPI) was 60mL/min/1.73m^2^ (standard deviation ±41mL/min/1.73m^2^) and most patients also had either proteinuria (86%) and/or hematuria (43%). The most common indication for kidney biopsy in our cohort was nephrotic syndrome (n=5, 33%), followed by acute or rapidly progressive kidney injury (n=3, 20%) and chronic kidney disease of unknown etiology (n=3, 20%).

### Pathological Findings

Pathological diagnosis and clinical presentation of patients are described in Tables 1 and 2. The most frequent diagnosis were chronic interstitial nephritis (CIN) (n=3) and AA amyloidosis (n=3), followed by IgAN (n=2) and focal segmental glomerulosclerosis (FSGS) (n=2). We also found 1 case of membranous nephropathy (MN) and 1 patient with immune complex-mediated membranoproliferative glomerulonephritis (IC-MPGN) attributed to AS. Kidney biopsy did not reveal significant alterations in two patients and there was insufficient material in another case not allowing a diagnosis. In the subsequent sections we describe 4 illustrative cases.

**Table 1:** Clinical presentation and final kidney biopsy diagnosis

N	Sex/ Age	Reason for biopsy	Serum creatinine (mg/dL)	Hematuria	24h proteinuria (g)	GFR (CKD-EPI, mL/min/1.73m^2^)	Duration of AS	Treatment	Diagnosis
1	M/44	AKI	4	No	3	18		*NA*	*FSGS*
2	M/40	NS	0.8	No	14.4	115	15 years	NSAIDs	AA amyloidosis
3	*M/NA*	AKI	*9.1*	+	4.5	*NA*	>15 years	NA	Insufficient material
4	F/50	CKD	2.2	No	2	27		NSAIDs + SSZ	CIN
5	F/21	NS	0.7	+	5	126	5 years	NSAIDs + PDN + MTX	AA amyloidosis
6	M/73	NS	3	+	12.2	21		*NA*	MN
7	F/42	Sub-nephrotic proteinuria	0.5	No	1.1	120		MTX + PDN	No significant alterations
8	M/53	Sub-nephrotic proteinuria	1.2	No	1.3	72		SSZ	No significant alterations
9	M/53	Nephrotic proteinuria	1	No	6	90		SSZ	FSGS
10	M/63	CKD	3	No	0.9	23	46 years	NSAIDs + SSZ	CIN
11	M/61	RPKI	HD	*NA*	*NA*	0	2 years	Infliximab	IgAN
12	F/29	CKD	1.3	No	0.329	57	8 years	SSZ	CIN
13	M/36	NS	1.75	+++	12	51	15 years	NSAIDs	IC-MPGN
14	M/29	Hematuria and sub-nephrotic proteinuria	1.26	+++	0.686	79	7 years	MTX, Adalimumab	IgAN
15	F/78	NS	1.3	+	10.4	42	10 years	MTX + PDN	AA Amyloidosis

AKI: Acute Kidney Injury; CIN: Chronic Interstitial Nephritis; CKD: Chronic Kidney Disease; F: Female; FSGS: Focal Segmental Glomerulosclerosis; GFR: Glomerular filtration rate; HD: hemodialysis; IC-MPGN: Immune complex-mediated Membranoproliferative Glomerulonephritis; IgAN: IgA Nephropathy; M: male; MN: Membranous Nephropathy; MTX: Methotrexate; NA: not available; NS: Nephrotic Syndrome; NSAIDs: Non-steroidal anti-inflammatory drugs; PDN: Prednisolone; RPKI: Rapidly Progressive Kidney Injury; SSZ: Sulfasalazine;

**Table 2:** Kidney biopsy results

N	Light Microscopy	Immunofluorescence	Conclusion
1	Four globally sclerotic glomeruli and 2 showing a segmental sclerotic lesion. Extensive tubular atrophy with thyroidization and tubular basement membrane thickening. Interstitium with diffuse fibrosis.	NA	FSGS
2	All glomeruli presented amyloid deposition. Tubular, interstitial, and vascular segments showed no significant alterations.	AA (+++)	AA amyloidosis
3	Cortical fragment without glomeruli representation.	Negative	Insufficient material
4	Four ischemic and globally sclerotic glomeruli. Diffusely fibrotic interstitium with many areas of tubular atrophy surrounded by an intense inflammatory infiltrate. Some arteries with moderately thickening of the intima.	Negative	CIN
5	All glomeruli presented amyloid deposition. Tubules, interstitium and vessels were normal.	AA (++)	AA amyloidosis
6	All glomeruli showed capillary wall thickening and spikes. Moderate interstitial fibrosis with chronic inflammatory infiltrate. Frequent tubular atrophy. Segmental hyalinosis in some arteries.	IgG (+)	MN
7	No significant alterations on the glomeruli, vessels and tubules. Small intersticial fibrous bands.	Negative	No appreciable alterations
8	Two globally sclerotic glomeruli with the remaining presenting small mesangial expansion; 3 hypertrophied glomeruli. Well preserved tubules and vessels. Limited areas of interstitial fibrosis with small mononuclear inflammatory infiltrate.	Negative	No appreciable alterations
9	One glomerulus with segmental sclerotic lesion, 3 ischemic glomeruli with thickened bowman capsules and the remaining being hypertrophied. Bands of interstitial fibrosis and tubular atrophy. No vascular changes reported.	C3 (+)	FSGS
10	Four ischemic and globally sclerotic glomeruli with the remaining presenting slight mesangial proliferation. Diffuse (90%) interstitial fibrosis with tubular atrophy and peritubular inflammatory infiltrate suggestive of granuloma formation. Arteries with intimal thinking, duplication of the internal elastica and arteriolar hyalinosis.	Negative	CIN
11	Diffuse endo and extra-capillary hypercelullarity with total cellular crescents with abundant neutrophil infiltration and necrotizing lesions in 5 glomeruli. Marked acute tubular necrosis and erythrocyte casts. Interstitial fibro-edema. No vascular alterations.	Negative	IgAN (M0E1S0T0-C2)
12	All glomeruli showed bowman capsule thickening without other alterations. 60% atrophic tubules. Marked interstitial fibrosis with inflammatory infiltrate without eosinophils or neutrophils.	Negative	CIN
13	Five globally sclerotic glomeruli with the remaining showing endocapillary hypercelullarity, lobulation, and double contours of the capillary wall, some with cellular crescents. Extensive interstitial fibrosis with acute inflammatory infiltrate and tubulitis. Tubular atrophy. Light vascular hyalinosis.	IgG (+++), C3 (+), kappa (+++), lambda (+++)	IC-MPGN
14	All glomeruli showed mesangial expansion and proliferation. Interstitial fibrosis in <5% of the fragment without inflammatory infiltrate. Tubules and vessels without appreciable alterations.	IgA (++), C3 (+), kappa (+), lambda (+)	IgAN (M1E0S0T0-C0)
15	Glomeruli presented amorphous deposition. Diffuse tubular atrophy.	AA (++)	AA Amyloidosis

CIN: Chronic Interstitial Nephritis; FSGS: Focal Segmental Glomerulosclerosis; IC-MPGN: Immune complex-mediated Membranoproliferative Glomerulonephritis; IgAN: IgA Nephropathy; MN: Membranous Nephropathy; NA: not available.

#### 1. CIN (Patient 10)

**Figure 1:**
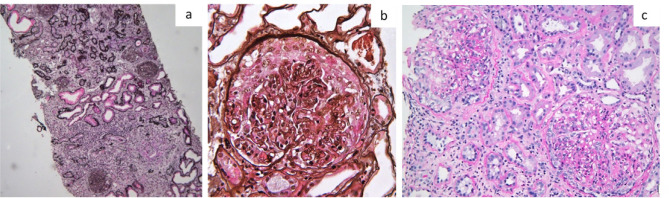
Kidney biopsy findings: a. Inflammatory infiltrate in an area of interstitial fibrosis and tubular atrophy consistent with Chronic Interstitial Nephritis (Patient 10, Silver stain, 6X); b. Cellular crescent, endocapillary proliferation and acute tubular necrosis in a patient with IgA Nephropathy (Patient 11, Silver Stain, 400X); c. Endo and extra-capillary proliferation in a patient with Membranoproliferative Glomerulonephritis (Patient 13, Periodic Acid Schiff stain, 200X).

A 63-year-old man with past medical history of AS diagnosed at 17 years of age, poorly controlled hypertension, and obesity was referred to the nephrology department for impaired kidney function with sCr of 1.6 mg/dL. Usual medication consisted in olmesartan, aliskiren, amlodipine, hydrochlorothiazide, fenofibrate, and allopurinol without recent changes or over-the-counter medications. Despite having controlled AS symptoms at the time of referral with no need for therapy, the patient had previous history of sulfasalazine and NSAIDs use. Aliskiren was suspended and carvedilol was initiated for better control of hypertension. Complementary studies revealed subnephrotic proteinuria (0.9g/24h), normal urinary sediment, and an erythrocyte sedimentation rate (ESR) of 56 mm/h. Immunologic studies and serum electrophoresis were unremarkable as were hepatitis B (HBV), C (HCV), and human immunodeficiency (HIV) virus serologies. For the next 6 months, the patient evolved with progressive kidney impairment reaching a sCr of 3 mg/dL. Kidney biopsy was performed and revealed 90% of interstitial fibrosis with tubular atrophy and an area of inflammatory infiltrate, consistent with CIN (Figure 1a). As other etiologies were excluded, CIN was attributed to chronic analgesic abuse and/or previous use of sulfasalazine. The patient progressed to kidney failure and eventually started peritoneal dialysis 2 years after the diagnosis.

#### 2. AA amyloidosis (Patient 15)

Recently, Domingos et al^4^ reported the case of a 78-year-old woman with a seronegative polyarthritis evolving for 10 years and corticoid-induced diabetes mellitus who presented to the nephrology department for NS (anasarca, sCr of 1.3 mg/dL, 10.4 g/24h of proteinuria, and hypoalbuminemia of 1.4 g/dL). She also complained of constitutional symptoms, diarrhea, and inflammatory arthralgia. Usual medication consisted in methotrexate, deflazacort, esomeprazole, tapentadol, folic acid, and calcium carbonate plus cholecalciferol and insulin. Despite the main complaint was of peripheral arthritis, the patient also described a lower back pain. Bilateral sacroiliitis on image studies together with a positive HLA-B27 antigen made the definitive diagnosis of AS. Complementary studies revealed elevated ESR (117 mm/h) and C-Reactive Protein (CRP) (77 mg/L) and slight microscopic hematuria. Occult neoplasia, infection, and autoimmunity were excluded and a kidney biopsy was performed. Light microscopy showed amorphous deposition in the mesangium and small arterioles of a material that was Congo-red positive and showed apple-green birefringence under polarized light. Immunofluorescence was positive for AA protein and the definite diagnosis of AA amyloidosis was made. Similarly, AA amyloidosis was also found on intestinal biopsy. As reported by the authors, etanercept was prescribed with improvement of kidney function and arthralgias.

#### 3. NIgA (Patient 11)

A 61-year-old man presented to the emergency department for macroscopic hematuria and reduced urinary output. He had previous medical history of diabetes and AS diagnosed 2 years before. Usual medication consisted in glibenclamide, metformin, and infliximab, without recent use of NSAIDs. The patient denied asthenia, dyspnea, cough, hemoptysis, or other relevant complaints including upper respiratory symptoms. On examination, he was afebrile, hypertensive, and anuric with peripheral edema, but without need for supplemental oxygen therapy. He did not show any signs of rash or cutaneous purpura. Lab test revealed AKI with sCr of 6.4 mg/dL and urea of 130 mg/dL. Serum electrolytes, CRP, and albumin were within normal range. Thorax x-ray showed mild pleural effusion and renal ultrasound excluded obstruction. Diuretic therapy was initiated without improvement and the patient was started on hemodialysis. Complementary investigation revealed negative ANCA, anti-glomerular basement membrane, ANA, and anti-dsDNA antibodies. Screening for hepatitis B, C, and HIV were negative, as were other microbiologic studies. Serum electrophoresis was unremarkable. Kidney biopsy was performed and revealed a globally proliferative glomerulonephritis with extensive endocapillary and extracapillary proliferation, with total cellular crescents in 8 of the 15 glomeruli and necrotizing lesions in five of them (Figure 1b). Tubules showed extensive acute tubular necrosis and erythrocyte casts, interstitium revealed only diffuse edema and there were no vascular alterations. Immunofluorescence revealed predominant IgA mesangial deposition and a crescentic IgAN was diagnosed. Endovenous cyclophosphamide and steroid treatment were initiated (methylprednisolone for 4 consecutive days followed by 1 mg/kg daily oral prednisolone). As the patient remained dialysis-dependent, plasmapheresis was started and a total of 5 sessions were performed without improvement. During the course of the hospitalization, the patient evolved unfavorably due to an infectious complication and eventually died from sepsis one month after admission.

#### 4. MPGN (Patient 13)

Finally, we describe a case of a 38 year-old man with end-stage CKD on peritoneal dialysis (PD). During PD follow-up, he presented with a constitutional syndrome characterized by progressive weight loss, low grade fever, inflammatory arthralgia, mainly of the lumbar joints but occasionally of the wrist and the elbow, polyserositis, and sustained elevated CPR. There were no complaints suggestive of infection including peritonitis, dialysis dose was adequate (Kt/V of 2), and the patient was euvolemic. Labs on admission were remarkable for normocytic normochromic anemia with hemoglobin of 7.4 g/dL, high ferritin levels (685 mg/dL), elevated ESR (87 mm/h), and CPR (140 mg/L). Extensive investigation for occult neoplasia and infectious diseases were all negative, as were ANA, anti-dsDNA, ANCA, and rheumatoid factor. Complement levels were normal.

CKD had been diagnosed two years before after the patient presented with NS (proteinuria of 12 g/24h, hypoalbuminemia of 2.3 g/dL), impaired kidney function (sCr 1.7 mg/dL), and hematuria. The patient also had a previous history of lower back pain starting at young age, chronic NSAIDs use (resumed when CKD was diagnosed), overweight, hypertension, and active smoking.

As an undiagnosed autoimmune disease was suspected, previous kidney biopsy was reviewed (Figure 1c). Light microscopy showed 12 globally sclerotic glomeruli. All of the remaining glomeruli presented endo and extra-capillary proliferation, lobulation, and double contours. There was also extensive interstitial fibrosis. IF studies on paraffin fragment showed predominant IgG, kappa, lambda, and mild C3 deposition on capillary wall and electron microscopy presented subendothelial IC deposits. As neoplastic and infectious diseases were excluded, the diagnosis of an autoimmune disease was pursued. Sacroiliitis was found on MRI studies and HLAB27 antigen returned positive, allowing for the diagnosis of AS with associated renal involvement by IC-mediated MPGN. The patient was started on adalimumab and evolved with improvement of articular complaints, resolution of the fever, and decreased inflammatory parameters.

## Discussion

Manifestations of kidney disease in AS may not be as rare as initially thought. Reported prevalence varies from 8^5^ to 35%^6^, depending on case series and mainly reflecting involvement by urinalysis alterations, although impaired kidney function has also been described^6,7^. Renal pathology, on the other hand, is largely unknow. Previous reviews report AA amyloidosis, followed by IgAN, as the most common histological findings in kidney biopsies of AS patients^7^. Since then, a small number of case series and some case reports have been published. Results are summarized in Table 3.

**Table 3:** Renal pathological alterations in patients with ankylosing spondylitis

Author (year)	Country	Biopsy-proven kidney disease (n)	Diagnosis (n)	Reference
He et al (2020)	China	62	IgAN (46) MCD/FSGS (3) IgAN + other GN (2) Idiopathic MN (2) Hypertensive nephropathy (2) Lupus nephritis (2) Non-specified proliferative GN (1) AA amyloidosis (1) Oxalate nephropathy (1) Glomerular minor lesion (1) Metabolic-related nephropathy (1)	11
Wu et al (2018)	China	21	IgAN (8) Non-specified proliferative GN (7) AIN (2) MN (2) Mesangioproliferative GN + interstitial nephritis (1) Mild pathological GN (1)	3
Champtiaux et al (2020)	France	20	IgAN (20)	13
Taarit et al (2005)	Tunisia	12	Non-specified type Amyloidosis (6) IgAN (3) FSGS (1) Purely endocapillary proliferation (1) Non-specified interstitial nephritis (1)	20
Barbouch et al (2018)	Tunisia	7	AA amyloidosis (7)	9
Lee et al (2012)	South Korea	6	IgAN (2) Chronic GN (2) AA Amyloidosis (1) Thin GBM (1)	4
Azevedo et al (2011)	Brazil	5*	IgAN (3) Thin GBM disease (1)	21
Corredor et al (2017)	Spain	1	AIN secondary to Adalimumab	10
Domingos et al (2019)	Portugal	1	AA amyloidosis	6
Gupta et al (2009)	India	1	MN	14
Kaushik et al (2011)	Albany	1	MN secondary to Etanercept	15
Chen et al (2017)	China	1	MN	16
Botey et al (1981)	Spain	1	MN	17
Alp et al (2013)	Turkey	1	FSGS	18
Tugsal et al (2019)	Turkey	1	FSGS secondary to Infliximab	19

AIN: Acute Interstitial Nephritis; CIN: Chronic Interstitial Nephritis; FSGS: Focal Segmental Glomerulosclerosis; GBM: Glomerular basement membrane; IC-MPGN: Immune complex-mediated Membranoproliferative Glomerulonephritis; IgAN: IgA Nephropathy; MCD: Minimal Change Disease; MN: Membranous Nephropathy;

* Spondyloarthritis patients (3/5 were ankylosing spondylitis patients.

Both AA amyloidosis and CIN were the most frequent diagnoses in our study. Regarding AA amyloidosis, evidence suggests that up to 6.1% of the AS patients may develop this complication following a 20-year period with a mean time from diagnosis of 14.4±10 years. High ESR has been reported as an independent predictive factor for renal AA amyloidosis^8^. In the present study, we highlight a case previously reported by Domingos et al^4^. The patient presented with nephrotic syndrome and ERS >100 mm/h at the time of kidney biopsy and was successfully treated with etanercept. Lee et al showed similar results^3^. Anti-Tumor Necrosis Factor (TNF)-α, together with NSAIDs, are currently the mainstay treatment for AS. We hypothesize that the reduced prevalence of AA amyloidosis in our series, compared with existing literature, may be due to a lower degree of inflammation in our patients and that further reduction of this diagnosis is expected in future cohorts due to novel and more efficient therapeutic regimens.

Although CIN is uncommon in other series, we do not find our results surprising. As previously discussed, NSAIDs are part of the first-line therapy for AS and are commonly used over a long period of time, being the only medication needed for many patients^1^. Furthermore, the association between the repeated use of NSAIDs and CIN is well established. Given its ubiquitous use in this particular disease, we hypothesize that even if we could not establish previous medications in some of the patients, the high prevalence of CIN in our series may be attributed to NSAIDs use (with or without contribution of other agents such as sulfasalazine). Nevertheless, we cannot completely exclude CIN as an uncommon renal manifestation of AS. More studies with larger cohorts are needed to investigate this possibility. Although we did not find a case of acute interstitial nephritis (AIN), there is one case report of adalimumab-associated AIN for treatment of AS^9^. As other biological agents are being studied and increasingly used, we urge clinicians to be highly suspicious of this diagnosis as well.

AS has long been described as a cause of secondary IgA nephropathy^7^. However, we believe this association should be interpreted with caution. As IgAN is the most common glomerulonephritis worldwide, we cannot exclude that this association occurs by chance. Moreover, we should be aware that the largest series of biopsy-proven kidney disease in AS were conducted in Asian populations where IgAN is particularly common^3,10,11^. In our series, IgAN was the third most frequent diagnosis. It has been suggested that downregulation of IgA Fc receptors (CD89) on blood phagocytes of AS patients leads to IgA immune-complex defective clearance and may explain more than a fortuitous association^12^. Interestingly, in a recent French nationwide study, spondyloarthritis (SpA)-associated IgAN was shown to have poorer prognosis compared to primary IgAN. These patients seem to present with faster decline in kidney function and greater prevalence of crescents on pathological examination^13^. The case described in the present study illustrates these results, where a crescentic IgAN was diagnosed in a Caucasian patient with AS. The reason of these findings is still unknown and deserves further investigation, although systemic inflammation mediated by TNF-α may play a role.

Other less commonly described pathological alterations in AS patients include MN^7,10,11,14-17^, FSGS^7,11,18-20^, and unspecified proliferative GN^7,10,11,20^. Given their rarity, it is difficult to establish causality. In our study, patients diagnosed with FSGS had no other known factors suggestive of a secondary cause. The only case of MN was diagnosed in 1999, before the anti-PLA2R era, and thus we cannot exclude a coincidental diagnosis of a PLA2R-associated MN. We also highlight a case of IC-MPGN. To our knowledge, this is the first case described in the literature.

The population analyzed in our cohort is somewhat different from the ones in previous series. Although we found a similar male predominance, we also showed that our patients were on average older at the time of kidney biopsy than previously reported. Considering that AS is commonly diagnosed in early adulthood, this may have impacted our results, as older age is probably associated with longer course of disease.

Our study has several limitations. The retrospective design with a long period of inclusion and multicentric origin of the patients hindered collection of all relevant clinical data. Besides, given the small number of patients, it was not possible to establish significant correlations. Thus, we present an observational study describing renal pathological alterations in AS patients for a 36-year period and present illustrative cases. Despite the limitations, this study is remarkable for being one of the largest series addressing this issue in Caucasian patients, together with the one from Taarit et al^20^. In contrast with their results, we found a higher prevalence of CIN and a lower prevalence of amyloidosis. Also, we found one case of IC-MPGN and one MN, pathological patterns not described in their series.

## Conclusion

Renal manifestations of AS include a heterogenous group of diseases that are poorly characterized. They may be related to the autoimmune disease or to treatment modalities. In the past, AA amyloidosis was the most common diagnosis. More recently, a number of case series showed an increasing prevalence of IgAN^10,11,21^, mainly in Asian cohorts. Moreover, it has been suggested that SpA-IgAN may be associated with poorer renal outcomes compared to primary IgAN in Caucasian patients. As renal abnormalities may contribute to long-term prognosis in a sizable portion of AS patients, larger prospective studies are needed to investigate kidney disease in AS.
